# Maternal vitamin D deficiency increases the risk of adverse neonatal outcomes in the Chinese population: A prospective cohort study

**DOI:** 10.1371/journal.pone.0195700

**Published:** 2018-04-24

**Authors:** Yuanliu Wang, Honghui Li, Min Zheng, Yubi Wu, Ting Zeng, Jinjian Fu, Dingyuan Zeng

**Affiliations:** 1 Department of Obstetrics and Gynecology, Liuzhou Maternity and Child Health Care Hospital, Liuzhou, Guangxi, China; 2 Liuzhou Key Laboratory of Children Developmental Disorders (Liuzhou Maternal and Child Health Care Hospital), Liuzhou, Guangxi, China; 3 Department of Pediatrics, Liuzhou Maternity and Child Health Care Hospital, Liuzhou, Guangxi, China; 4 Department of Laboratory Medicine, Liuzhou Maternity and Child Health Care Hospital, Liuzhou, Guangxi, China; University of Missouri Columbia, UNITED STATES

## Abstract

**Background:**

Although vitamin D (vitD) deficiency is a common problem in pregnant women, in China, few studies have focused on the relationship between maternal vitD deficiency throughout the three trimesters and subsequent neonatal outcomes in China.

**Methods:**

Between 2015 and 2016, maternal serum and neonate cord blood samples were collected from 1978 mother-neonate pairs from Liuzhou city.

**Results:**

The mean concentrations of 25-hydroxy vitD (25(OH)D) were 16.17±6.27 and 15.23±5.43 ng/ml in the mother and neonate groups, respectively, and the prevalence values of vitD deficiency in the two groups were 78.18% and 83.27%, respectively. Logistic regression showed that maternal vitD deficiency independently increased the risk of gestational diabetes mellitus (GDM) (adjust OR, aOR 1.08; *P* = 0.026). A relatively lower risk of vitD deficiency was observed in the third trimester than in the first and second trimester (aOR 0.80; *P* = 0.004). VitD-calcium cosupplementation during pregnancy improves the vitD deficiency in both the maternal and neonatal groups (aOR 0.56, 0.66; *P*<0.001 and 0.021, respectively). Maternal vitD deficiency significantly increased the risk of neonatal low birth weight (LBW) (aOR 2.83; *P* = 0.005) and small-for-gestational-age (SGA) (aOR 1.17; *P* = 0.015). There was a positive correlation between maternal and neonatal vitD deficiency (*r* = 0.879, *P*<0.001). VitD supplementation during pregnancy significantly reduced the risk of giving birth to LBW infants (OR = 0.47, 95%CI = 0.33–0.68, *P*<0.001).

**Conclusions:**

Further research focusing on the consumption of vitD with calcium during pregnancy and the consequential clinical outcomes in Chinese pregnant women is warranted.

## Introduction

Vitamin D (vitD) is a well-known secosteroid hormone for its classical functions, such as skeletal health and bone metabolism[1]. Recently, there has been considerable recognition of the importance of its role in modifying the immune system and regulating cell proliferation and cell differentiation[2,3]. VitD deficiency was defined as a serum 25-hydroxy vitD (25-OH D) concentration less than 20 ng/ml, while vitD insufficiency was defined as a 25-(OH)-D concentration less than 30 ng/ml[4]. Due to the function of vitD, many concerns have been raised regarding important impacts of vitD deficiency and the association risk of diseases such as chronic kidney disease[5], cystic fibrosis[6], obesity[7], etc.

An abundance of epidemiological evidence links vitD deficiency and insufficiency to a variety of adverse maternal and neonatal outcomes including preeclampsia, hypertension, gestational diabetes mellitus (GDM), spontaneous abortion, intrauterine growth restriction (IUGR), small size for gestational age (SGA), low birth weight (LBW) and premature birth[8–13]. It was reported that maternal vitD deficiency is considered an important biomarker which can change the glucocorticoid-related parameters in placenta. Maternal vitD deficiency induces the placental and fetal glucocorticoid exposure thus leads to the adverse outcome of fetal growth restriction eventually[14]. However, other observational studies have shown no association between the vitD status and adverse pregnancy outcomes[15,16].

Although there is growing evidence that vitD deficiency and insufficiency are associated with negative pregnancy and infant outcomes, evidence for the correlation of hypovitaminosis D with the potential risk to maternal-neonatal pairs is limited. The objective of the present study was to explore the correlation of maternal-neonatal pairs with vitD status and determine whether maternal vitD deficiency may increase the risk of adverse neonatal outcomes.

## Subjects and methods

### Study design and subjects

The Liuzhou Birth Cohort Study was a prospective population-based study with a birth cohort that recruited 2000 pregnant women between 2015 and 2016 at Liuzhou Maternity and Child Health Care Hospital. The inclusion criteria were mother-singleton-offspring pairs for whom serum samples from mothers and umbilical cord blood samples from neonates were obtained. Because there were reports[14,15] described the association between vitD deficiency and the risk of GDM, so this cohort included GDM. The exclusion criteria were maternal and neonatal endocrine disorders, such as diabetes mellitus (type 1 or 2) except GDM, thyroid disease, renal and cardiovascular disease, and a history of spontaneous abortion. Eight pregnant women who gave birth to twins, four women who had abortions, two women who had fetal deaths, and eight women who withdrew were excluded from this study. A total of one thousand nine hundred and seventy eight mother-singleton-offspring pairs were included in this study.

### Ethics statement

The study was conducted in accordance with the principles of the Declaration of Helsinki and was approved by the Ethics Committee of Liuzhou Maternity and Child Health Care hospital. In addition, all the participants were given written informed consent in the study. All procedures and methods involving human samples were in accordance with approved guidelines.

### Measurement of the serum 25(OH)D level

Blood samples were collected and sent to the clinical laboratory for the vitD measurement. Serum 25(OH)D levels were measured using an electrochemiluminescence immunoassay (ECLIA) kit (Roche Elecsys 10100/201 system, Germany) according to manufacturer’s instructions as described previously [17].

### Definitions

Season was defined by the following groups: spring (March to May), summer (June to August), autumn (September to November), winter (December to February).

SGA was calculated as birth weight below the 10^th^ percentile for gestational age[18].

IUGR was defined as a failure of the fetus to achieve its optimal growth potential according to the criteria adopted by the American College of Obstetricians and Gynecologists (ACOG)[19].

Preeclampsia was defined as the presence of hypertension (systolic blood pressure ≥140mmHg and/or diastolic blood pressure ≥90mmHg) after 20 weeks of gestation with the presence of proteinuria (≥0.3g/24h or ≥1+ on dipstick) in previously normotensive women, according to the criteria adopted by ACOG[20].

A 75g oral glucose tolerance test (75g OGTT) was performed between 24–48 weeks of gestation. GDM was defined as the fasting plasma glucose ≥92mg/dl (5.1mmol/L) or 1h plasma glucose ≥180mg/dl (10.0mmol/L), or 2h plasma glucose ≥153mg/dl (8.5mmol/L), according to the recommendations of reference[21].

Weight of birth was measured within 15 minutes after birth. Low birth weight was defined as those newborns with birth weight less than 2500g.

Minority ethnic group: There are 56 different ethnic groups in China. Han and Zhuang are the two major ethnic groups in Guangxi, which consist of about 90% of the population in Guangxi. The minority ethnic group is defined as any other ethnic groups except Han and Zhuang.

### Data collection

VitD status based on laboratory and clinical records from two electronic databases was reviewed. The following data were extracted: maternal age, pre-pregnancy BMI, gestational age, season of blood draw, trimester, GDM, preeclampsia, vitD supplemental during pregnancy, vitD-calcium cosupplementation during pregnancy, birth weight, infant gender, preterm delivery, IUGR, SGA, neonatal/maternal serum 25(OH)D levels

### Statistical analysis

SPSS software version 20.0 (SPSS Inc. Chicago, IL, USA) and R (http://www.R-project.org) were used for data analyses. Nonparametric methods were used to compared variables of which were not normally distributed. The Kruskal-Wallis rank test was used to compare the differences between trimesters, seasons of maternal blood draw, birth season, and of 25(OH)D measurements. Comparisons of associations of the 25(OH)D level with different groups of related factors were performed using univariate analysis methods. The following variables were entered as predictors in the model: maternal age, pre-pregnancy BMI, gestational age, season of blood draw, trimester, GDM, preeclampsia, vitD supplemental during pregnancy, vitD-calcium cosupplementation during pregnancy, birth weight, infant gender, preterm delivery, IUGR, SGA, neonatal/maternal vitD deficiency. All variables with *P*<0.10 were selected for inclusion in the multivariate logistic regression model to identify predisposing risk factors that are associated with maternal and neonatal vitD deficiency. *P* values of <0.05 were considered significant. All *P* values were corrected by Bonferroni’s method for multiple testing.

## Results

### 25(OH)D concentrations

Table 1 shows the quartile values of 25(OH)D (Q1 = 11.92 ng/ml, Q2 = 14.84 ng/ml, and Q3 = 18.85 ng/ml) of the maternal vitD status and (Q1 = 11.40 ng/ml, Q2 = 14.38 ng/ml, and Q3 = 18.01 ng/ml) of the neonatal vitD status. Maternal-neonatal pairs of participants had a median cord blood 25(OH)D concentration of 16.17 ng/ml and 15.23 ng/ml, respectively. Overall, 79.18% and 83.27% of respective Liuzhou maternal-neonatal participant pairs had serum 25(OH)D levels <20 ng/ml and 96.41% and 97.98% had levels <30 ng/ml (Table 1).

**Table 1:** VitD level and the prevalence of vitD deficiency in maternity and neonates (n = 1978).

Variable	Maternal 25(OH)D	Neonatal 25(OH)D
Q1	11.92	11.40
Q2	14.84	14.38
Q3	18.85	18.01
Q4	53.80	47.04
Min (ng/ml)	1.28	2.68
Max (ng/ml)	53.80	47.04
Mean±SD (ng/ml)	16.17±6.27	15.23±5.43
The prevalence of vitD deficiency (%) [25(OH)D<20 ng/ml]	79.18	83.27
The prevalence of vitD insufficiency (%) [<30(ng/ml)]	96.41	97.98

Q: quarter

### Maternal characteristics and vitD status

**Fig 1:**
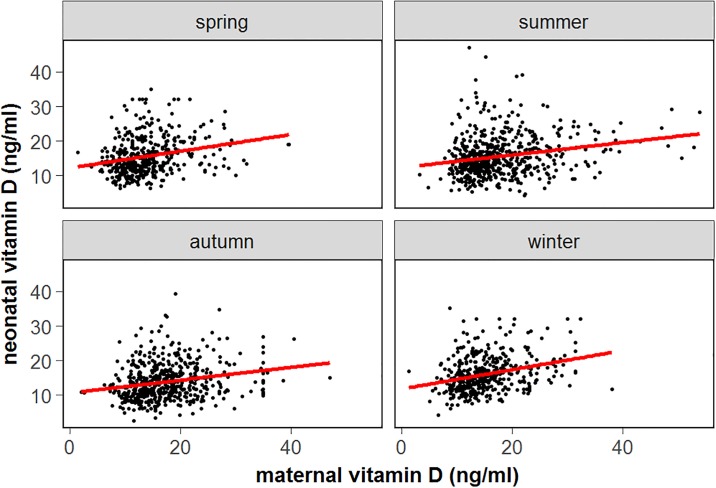
Association between maternal 25(OH)D level during pregnancy and neonatal cord blood 25(OH)D level distributed by maternal blood drew season.

**Fig 2:**
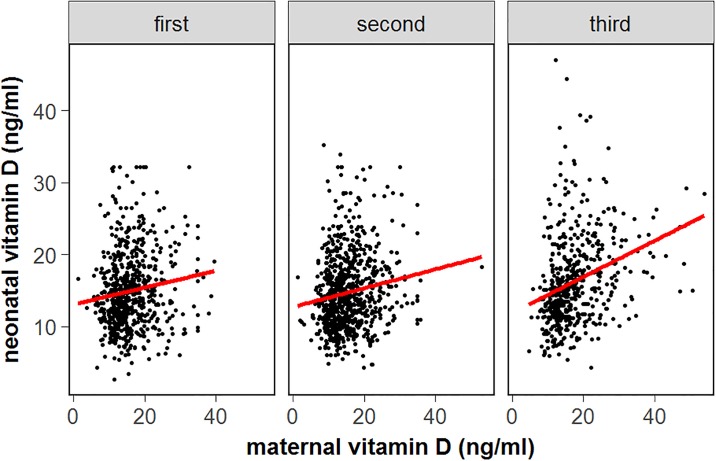
Association between maternal 25(OH)D level during pregnancy and neonatal cord blood 25(OH)D level distributed by trimester.

Evaluation of the potential risk factors explored in this study revealed that season was a determinant of maternal vitD status. Compared to spring, a significant increase in maternal 25(OH)D concentrations was observed in the summer, autumn and winter seasons; the odds ratio (OR) and 95% confidential interval (95% CI) were 0.31 (0.22–0.44), 0.33 (0.23–0.48), and 0.60 (0.40–0.90), respectively (Table 2, Fig 1). The maternal 25(OH)D concentration was significantly higher in the third trimester than in the first two trimesters; the OR values (95% CI) were 1.52 (1.15–1.99) and 1.54 (1.17–2.04), respectively (Table 2, Fig 2). In the multivariate regression model, maternal vitD deficiency independently increased the GDM risk, the OR (95% CI) was 1.08 (1.04–1.10) (*P* = 0.026) (Table 3). A relatively lower risk of vitD deficiency was observed in the third trimester than in the first and second trimester (adjusted OR = 0.80, 95% CI, 0.69–0.93; *P* = 0.004). Maternal 25(OH)D concentration was inversely associated with SGA risk, the OR (95% CI) was 1.17 (1.03–1.32) (*P* = 0.015).

**Table 2:** The correlations of independent factors with 25(OH)D in maternity by univariate analysis.

Variable		OR	95%CI	*P* value
Maternal age(years)				0.005
<30	1179(59.61)	Reference		
30–34	534(26.99)	0.78	0.60–1.02	
≥35	265(13.40)	1.40	1.04–1.90	
Maternal prepregnancy BMI				0.705
<23	1445(73.05)	Reference		
≥23	533(26.85)	0.97	0.75–1.25	
Gestational age (weeks)				0.701
<37	101(5.1)	Reference		
37–39	1073(54.25)	1.08	0.76–1.32	
40+	804(40.64)	1.07	0.86–1.35	
Ethnic				0.716
Han	1006(50.8)	Reference		
Zhuang	795(40.2)	1.07	0.75–1.23	
Minority	177(9.0)	1.41	0.83–1.56	
Season of blood draw				0.001
Spring	435(21.99)	Reference		
Summer	594(30.03)	0.31	0.22–0.44	0.001
Autumn	533(26.85)	0.33	0.23–0.48	0.001
Winter	416(21.03)	0.60	0.40–0.90	0.014
Trimester				0.002
First	652(32.96)	1.52	1.15–1.99	0.003
Second	804(40.65)	1.54	1.17–2.04	0.001
Third	522(26.39)	Reference		
GDM				<0.001
No	1811(91.6)	Reference		
Yes	167(8.4)	1.06	1.03–1.09	
PE				0.302
No	1915(96.8)	Reference		
Yes	63(3.2)	0.98	0.94–1.02	
vitD supplemental during pregnancy				0.001
No	878(44.39)	Reference		
≤6 times/week	676(34.18)	0.47	0.36–0.61	0.001
≥1 time/day	424(21.43)	0.15	0.23–0.41	0.001
vitD-calcium cosupplementation during pregnancy				0.001
No	858(43.38)	Reference		
≤6 times/week	717(36.25)	0.43	0.33–0.56	0.001
≥1 time/day	403(20.37)	0.29	0.22–0.39	0.001
Birth weight (g)				0.001
<2500	130(6.57)	2.75	1.32–3.01	
≥2500	1848(93.43)	Reference		
Gender				0.497
Boy	1046(52.88)	Reference		
Girl	932(47.12)	0.93	0.75–1.15	
Preterm delivery (<37 weeks)				0.672
No	1839(92.97)	Reference		
Yes	139(7.03)	0.91	0.59–1.41	
IUGR				0.106
No	1941(98.1)	Reference		
Yes	37(1.9)	0.95	0.89–1.01	
SGA				0.012
No	1800(91.0)	Reference		
Yes	178(9.0)	1.17	1.03–1.32	
Neonatal vitD deficiency (<20ng/ml)				0.001
No	331(16.73)	Reference		
Yes	1647(83.27)	1.06	1.04–1.08	

BMI: body mass index; GDM: gestational diabetes mellitus; PE: preeclampsia; IUGR: intrauterine growth restrictions; SGA: small-for gestational-age

**Table 3:** The correlations of independent factors with 25(OH)D in maternity by multivariate analysis.

Late Trimester	0.80	0.69–0.93	0.004
Gestational diabetes mellitus	1.08	1.04–1.10	0.026
vitD-calcium cosupplementation during pregnancy	0.56	0.48–0.65	<0.001
Small-for gestational-age	1.17	1.03–1.32	0.015
Neonatal vitD deficiency	1.92	1.47–2.49	<0.001

VitD and vitD-calcium cosupplementation during pregnancy significantly reduced the risk of maternal vitD deficiency; the relative risks were 0.47 (95% CI, 0.36–0.61) and 0.15 (95% CI, 0.23–0.41) for vitD supplementation ≤6 times/week and ≥1 time/day, respectively. The same result was observed for vitD-calcium cosupplementation; for supplementation ≤6 times/week, the OR (95% CI) was 0.43 (0.33–0.56). For supplementation ≥1 time/day, this risk was reduced to 0.29 (95% CI, 0.22–0.39) compared to the group that did not take supplements. In the multivariate regression analysis, vitD-calcium cosupplementation during pregnancy increased the maternal vitD status; the OR (95% CI) was 0.56 (0.48–0.65) (*P* = <0.001) (Table 3).

**Fig 3:**
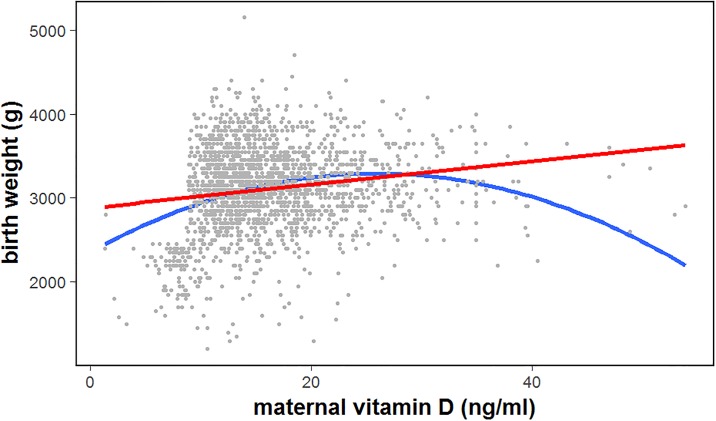
Scatter plot of correlation between maternal 25(OH)D level during pregnancy and neonatal birth weight (*r* = 0.522, *P*<0.001). Both the estimated regression line (red line) and a true curve line (blue line) demonstrate the inverse association between these two variables.

There was a positive correlation between the maternal vitD concentrations and neonatal birth weight (*r* = 0.522, *P*<0.001) (Fig 3). Maternal vitD deficiency significantly increased the risk of a low neonatal birth weight; the relative risk was 2.75 (95% CI, 1.32–3.01). Maternal vitD deficiency was an independent risk factor for neonatal vitD deficiency; the OR (95% CI) was 1.92 (1.47–2.49) (*P*<0.001).

### Neonatal characteristics and VitD status

**Fig 4:**
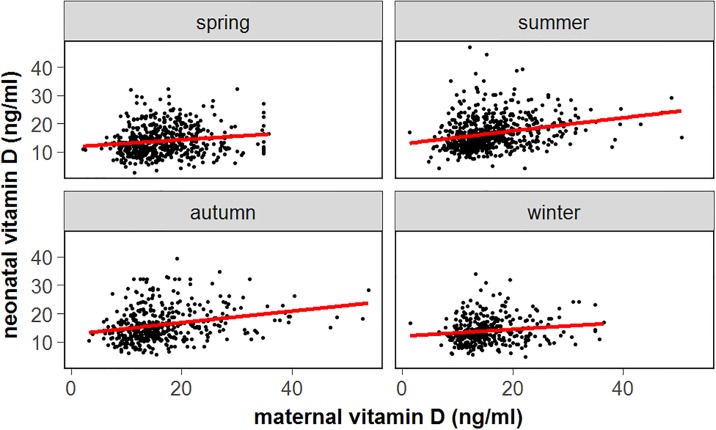
Association between neonatal cord blood 25(OH)D level and maternal 25(OH)D level during pregnancy distributed by birth season.

The neonatal cord blood 25(OH)D was lower in birth seasons of spring and winter (*P* trend <0.001) (Table 4, Fig 4), but this relationship was no longer significant in the multivariate regression model. VitD and vitD-calcium cosupplementation during pregnancy significantly reduced the neonatal vitD deficiency risk; the relative risks were 0.47 (95% CI, 0.36–0.61) and 0.15 (95% CI, 0.23–0.41) for vitD supplementation ≤6 times/week and ≥1 time/day, respectively, and the relative risks were 0.43 (95% CI, 0.33–0.56) and 0.29 (95% CI, 0.22–0.39) for vitD-calcium cosupplementation ≤6 times/week and ≥1 time/day, respectively (Table 4). In the multivariate regression analysis, vitD-calcium cosupplementation was a protective factor associated with neonatal vitD deficiency; the OR (95% CI) was 0.66 (0.46–0.94) (*P* = 0.021) (Table 5).

**Table 4:** The correlations of independent factors with cord blood 25(OH)D in neonate by univariate analysis.

Variable	n(%)	OR	95%CI	*P* value
Maternal age(years)				0.004
<30	1179(59.61)	Reference		
30–34	534(26.99)	1.18	0.89–1.55	0.243
≥35	265(13.40)	1.73	1.25–2.39	0.001
Maternal prepregnancy BMI				0.530
<23	1445(73.05)	Reference		
≥23	533(26.85)	0.96	0.83–1.10	
Gestational age (weeks)				0.151
<37	101(5.1)	Reference		
37–39	1073(54.25)	1.02	0.77–1.25	0.167
40+	804(40.64)	1.21	0.89–1.13	0.138
Ethnic				0.856
Han	1006(50.8)	Reference		
Zhuang	795(40.2)	0.91	0.87–1.04	
Minority	177(9.0)	1.05	0.93–1.21	
Birth season				0.001
Spring	549(27.76)	Reference		
Summer	653(33.01)	0.50	0.36–0.69	0.001
Autumn	425(21.49)	0.50	0.35–0.71	0.001
Winter	351(17.74)	0.90	0.59–1.38	0.641
GDM				0.778
No	1811(91.6)	Reference		
Yes	167(8.4)	0.99	0.97–1.03	
PE				0.196
No	1915(96.8)	Reference		
Yes	63(3.2)	1.03	0.98–1.07	
vitD supplemental during pregnancy				0.003
No	878(44.39)	Reference		
≤6 times/week	676(34.18)	0.63	0.48–0.82	0.010
≥1 time/day	424(21.43)	0.71	0.52–0.97	0.030
vitD-calcium cosupplementation during pregnancy				0.001
No	858(43.38)	Reference		
≤6 times/week	717(36.25)	0.62	0.48–0.82	0.001
≥1 time/day	403(20.37)	0.61	0.44–0.83	0.002
Birth weight (g)				0.002
<2500	130(6.57)	3.23	1.56–6.67	
≥2500	1848(93.43)	Reference		
Gender				0.714
Boy	1046(52.88)	Reference		
Girl	932(47.12)	1.05	0.83–1.32	
Preterm delivery (<37 weeks)				0.862
No	1839(92.97)	Reference		
Yes	139(7.03)	1.04	0.66–1.64	
IUGR				0.264
No	1941(98.1)	Reference		
Yes	37(1.9)	0.96	0.90–1.03	
SGA				0.215
No	1800(91.0)			
Yes	178(9.0)			
Maternal vitD deficiency (<20ng/ml)				0.001
No	412(20.83)	Reference		
Yes	1566(79.17)	2.03	1.56–2.64	

BMI: body mass index; GDM: gestational diabetes mellitus; PE: preeclampsia; IUGR: intrauterine growth restrictions; SGA: small-for gestational-age

**Table 5:** The correlations of independent factors with cord blood 25(OH)D in neonate by multivariate analysis.

Variable	OR	95%CI	*P* value
VitD-calcium cosupplementation during pregnancy	0.66	0.46–0.94	0.021
Low birth weight	2.83	1.36–5.89	0.005
Maternal vitD deficiency	1.79	1.36–2.35	0.001

**Fig 5:**
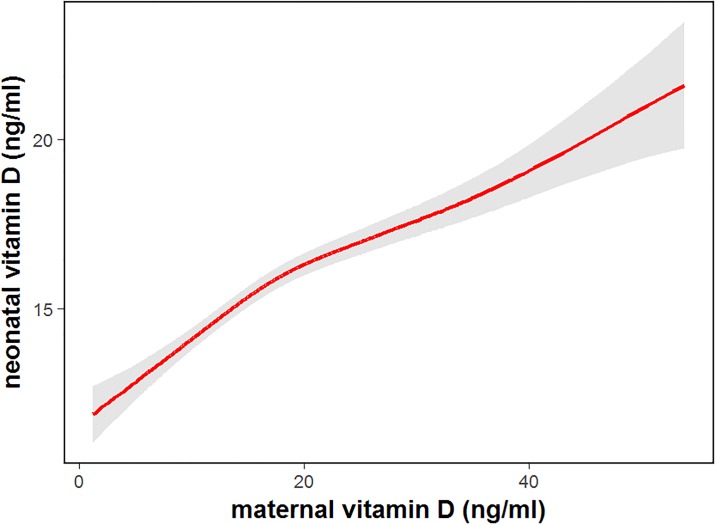
Neonatal cord blood 25(OH)D level was predicted by maternal 25(OH)D level modeled by restricted cubic splines. The solid line is the predicted cord blood level, the gray shading is 95% confidence intervals.

One hundred and thirty (6.57%) neonates had a LBW. The risk of vitD deficiency was much higher in neonates who had LBW than those who had a normal birth weight; the OR (95% CI) was 2.83 (1.36–5.89) (*P* = 0.005) (Table 5). The maternal vitD deficiency was also an independent risk factor for neonatal vitD deficiency; the OR (95% CI) was 1.79 (1.36–2.35) (*P*<0.001). There was a positive correlation between maternal and neonatal vitD deficiency (r = 0.879, *P*<0.001). The maternal vitD deficiency was a predictor for neonatal vitD deficiency, and the predictive model is shown in Fig 5. Maternal vitamin D supplementation played an important role in prevention of giving birth to LBW infants. In the vitD supplementation group, only 4.5% (50 women) gave birth to LBW infants, while 9.1% (80 women) who didn’t have vitD intervention gave birth to LBW infants. VitD supplementation during pregnancy significantly reduced the risk of giving birth to LBW infants (OR = 0.47, 95%CI = 0.33–0.68, *P*<0.001).

## Discussion

The recent establishment of a causal relationship between maternal vitD deficiency and adverse pregnancy outcomes is a matter of great public health concern[22–25]. The prevalence of maternal or neonatal vitD deficiencies has been extensively studied worldwide[8–10]. Few studies have been conducted on the vitD levels of both mothers and their newborns[11–16,21]. Our cohort study contains 1978 pairs of maternal-neonatal vitD samples and revealed that 79.18% of pregnant women and 83.27% of newborns had 25(OH)D levels below 20 ng/ml; for 25(OH)D levels below 30 ng/ml, which indicates vitD insufficiency, this figure increased to 96.41% in pregnant women and 97.98% in their newborns residing in Liuzhou, Western China. Although few studies have reported on the prevalence of the vitD status in pregnant women or newborns in China, their data agreed with ours, which indicated that a high prevalence of vitD deficiency occurred in both groups[26–29].

GDM was the most common maternal complication with the prevalence of 4.1%-27.5% globally[30]. We observed a significant relationship between 25(OH)D deficiency and the risk of GDM. Our study is consistent with others indicating that there was an inverse association between 25(OH)D and risk of GDM[15]. Two nested case-control studies using a prospective designed confirmed that lower vitD status was associated with a significantly increased risk of subsequent GDM[31,32], one of which provided further evidence showed that 25(OH)D concentrations below the top quartile (<73.5 mmol/L) increased a 2-fold greater risk of GDM during pregnancy[32]. Our study, along with worldwide cohort studies[28,29] suggested that vitD may play a role in glucose tolerance, which will enable the design of proper interventions (e.g., vitD supplementation) to reduce the rates of some maternal complications.

Most of the recently published randomized controlled trials (RCTs) have focused on whether maternal vitD supplementation may influence maternal or neonatal vitD deficiency[33,34]. Few studies have focused on the positive maternal and fetal outcomes of consuming vitD with calcium during pregnancy[34]. Emerging evidence suggests that vitD sufficiency has a positive impact on the skeletal system and women who simultaneously supplement vitD and calcium during pregnancy may have a reduced risk of adverse outcomes while improving fetal growth[34,35]. The recently published meta-analysis with pooled 13 RCTs also confirmed that maternal vitD supplementation can increase circulating 25(OH)D levels, birth weight and birth length[36]. Our study showed a corresponding increase in the serum 25(OH)D and cord blood 25(OH)D levels with increasing use of vitD-calcium cosupplementation in pregnant women from Liuzhou. Our findings were further supported by Park et al[37], who found that there are higher maternal concentrations of vitD biomarkers in pregnant women who simultaneously supplement vitD and calcium in their third trimester to ensure sufficient calcium delivery to the fetus. As vitD deficiency is a common problem in both pregnant women and infants, our study highlights the positive effect of vitD-calcium cosupplementation during pregnancy on both the maternal and neonatal vitD levels.

VitD plays an important role in fetal growth due to its key interaction with Ca^2+^ homeostasis and parathyroid hormones. A significantly increased risk of low neonatal birth weight among populations with vitD deficiency during pregnancy is anticipated and consistent with existing trends in the literature[38]. Growing evidence has indicated that maternal vitD deficiency may impair fetal growth and lead to a series of adverse pregnancy outcomes, including preterm delivery, IUGR, SGA and neonatal LBW[11–16]. VitD deficiency during pregnancy was associated with nearly 2-fold greater odds of a LBW in our study. In agreement with our study, a significant positive association between maternal vitD deficiency and LBW has been reported in an Iranian population[38]. Our study showed that lower vitD status was associated with a significantly increased risk of subsequent delivered SGA neonates. In another study conducted in Caucasians, 25(OH)D concentrations were significantly lower in mothers who delivered neonates who were SGA[11], which was consistent with our results.

Our study reported that maternal vitD deficiency has a positive correlation with neonatal vitD deficiency, and maternal vitD deficiency significantly increased the risk for neonatal vitD deficiency, while the relative risk was nearly 2-fold. Reports from another Chinese population demonstrated similar results. A study conducted in Beijing indicated that neither mothers nor newborns had normal levels of 25(OH)D (>30 ng/ml) and that a significantly positive correlation between maternal and neonatal 25(OH)D concentrations has been demonstrated (r = 0.89, *P*<0.001). Meanwhile, severe maternal vitD deficiency was associated with a higher risk of LBW neonates[39], which was consistent with our results. The Anhui birth cohort study showed that the relative risk for a LBW in the maternal deficiency group was 12.31[40].

The data from our study indicated that pregnant women in the third trimester had higher vitD concentrations. VitD deficiency occurred in both the maternal and neonatal groups. A high prevalence of vitD deficiency in both groups was associated with low neonatal birth weight. The successful concurrent use of vitD and calcium contributed to high vitD levels in the maternal and neonatal populations.

## Conclusions

In summary, our findings demonstrate the importance of interventions to ensure an adequate vitD level in pregnant women. Strategies should focus on different risk factors, and there should be continued efforts to increase the supplement with vitD and calcium during pregnancy which could in turn reduce the prevalence of vitD deficiency in both the maternal and neonatal groups.

## Supporting information

S1 File Checklist and IRB. (RAR)
